# Molding the Pain into Porcelain: The Silent Resilience of Arthritic Hands in Hong Kong’s Ceramic Studios

**DOI:** 10.3390/healthcare14081069

**Published:** 2026-04-17

**Authors:** Alice Yip, Wai Ping Cecilia Li Tsang, Jeff Yip, Chi Kong Calvin Yip, Man Ho Tim Li, Zoe Tsui, Ka Man Rachel Yip, Ka Wing Gavin Lee, Shuk Yu Maria Hung

**Affiliations:** 1 S.K. Yee School of Health Sciences, Saint Francis University, 2 Chui Ling Lane, Tseung Kwan O, New Territories, Hong Kong, China; ztsui@sfu.edu.hk (Z.T.); kmyip@sfu.edu.hk (K.M.R.Y.); syhung@sfu.edu.hk (S.Y.M.H.); 2 Hong Kong Arthritis & Rheumatism Foundation, Hong Kong, China; ceci4306@gmail.com (W.P.C.L.T.); gavinlee@netvigator.com (K.W.G.L.); 3 School of Nursing, Tung Wah College, Hong Kong, China; jeffreyyip@gmail.com; 4 School of Medical and Health Sciences, Tung Wah College, Hong Kong, China; calvinyip@twc.edu.hk; 5 Department of Psychiatry, Faculty of Medicine, The Chinese University of Hong Kong, Hong Kong, China; manholi@cuhk.edu.hk

**Keywords:** ceramics, hand exercises, disease burden, pain, RA, resilience, rheumatoid arthritis

## Abstract

**Background/Objectives:** Globally, rheumatoid arthritis (RA) patients struggle to meet the expectation of being active in their daily lives. The burden on these individuals is twofold, including physical limitations and emotional stress, which make looking after themselves a major challenge. Supporting self-management requires more than just offering strategies; it requires fitness. Whether we are proposing new daily habits or creative outlets like ceramic workshops, it is essential to tap into the patient’s perspective to understand exactly what kind of support will resonate with them. This study explored how ceramic workshops can help people with RA to build resilience and improve their well-being. **Methods:** Using a phenomenological study design, we interviewed 16 patients with RA in Hong Kong who engaged in ceramic workshops. These participants were selected through purposive sampling, and their insights were gathered via semi-structured interviews. We applied Colaizzi’s seven-step method to analyze the findings. **Results:** Four key themes emerged: (i) embodied manageability; (ii) clear comprehension of body limits; (iii) the meaningfulness of creating art; and (iv) supporting resilience. **Conclusions:** This study reveals that effective self-management support should prioritize patient-driven needs, particularly peer interaction and high-demand creative pursuits such as ceramic workshops.

## 1. Introduction

Rheumatoid arthritis (RA) is a long-term condition where the body’s immune system mistakenly attacks its own joints [1,2], causing significant inflammation, swelling, and pain [1]. While it can affect the whole body, it is often most damaging to the hands, making simple tasks that require limb and finger movement difficult. This condition is a global health concern, but it has a specific impact on the Chinese population in regions like Hong Kong (HK) [3,4]. Research has indicated that the prevalence of RA in China is approximately 0.28% to 0.4% of the population [5,6,7]. When looking especially at HK, studies suggest that about 0.35% of adults have this disease [7,8]. While specific data for HK and Macau are often combined with broader regional statistics, these areas face comparable challenges due to shared genetic environmental factors across the Greater Bay Area. For these patients, the loss of hand ability is not simply a physical symptom; it also severely impacts their everyday independence and quality of life (QoL) [9].

Medication is the first line of defense for people living with RA, which is needed to control pain and inflammation [10,11]. However, medication alone often is not enough to handle the full impact of the disease. To truly care for the whole person, not just their joints, physicians and researchers increasingly recommend including psychological and social support in the treatment plan [12,13,14,15]. There is strong evidence behind this holistic approach, which emphasizes building mental resilience and keeps patients physically active [16,17,18,19]. Up to 40% of RA patients experience anxiety or depression, which studies indicate can intensify their physical pain [20,21,22]. Furthermore, studies have shown that psychological interventions, such as cognitive behavioral therapy or self-management education, can enhance a patient’s ability to cope with pain by approximately 20% to 30% [9]. By combining medicine with strategies that improve mental toughness and encourage movement, patients can attain a better QoL than they would with medication alone.

Ceramic is essentially physical; the clay demands both fine motor precision and significant hand strength to manipulate [23,24]. Working with ceramics helps people connect their past and present experiences; it also provides a way to cope with and adjust to losing physical movement, particularly through ceramics activities tailored to those physical changes [25]. A report by the World Health Organization’s Health Evidence Network indicates that art forms such as ceramics play a significant role in promoting overall well-being, helping to prevent various mental and physical health issues, and aiding in the treatment and management of existing conditions [26]. Participating in creative expression fosters patient resilience and contributes to better clinical outcomes [27,28]. Ceramics, specifically, is often readily adopted by patients and has a longstanding history as a purposeful activity in rehabilitation therapy [29,30]. For individuals with RA, these requirements present a contradiction, as the craft is both a physically demanding challenge and a unique, physical approach to rehabilitation, providing a sense of satisfaction and creative self-expression [23,24,31,32]. Therefore, a practicable alternative to ceramics should be provided while maintaining the core principles of person-centered health interventions [33].

### Theoretical Framework

Rheumatoid Arthritis is frequently characterized in studies by its deficits, including the deterioration of joints, the continuity of pain, and the gradual loss of physical function [1,2]. While these clinical realities are obvious, focusing solely on pathology fails to capture the complex ways in which individuals with RA manage their lives to find stability and purpose. To address this issue, this study incorporates Aaron Antonovsky’s framework of Salutogenesis. This specific theory was selected because it represents a vital shift in investigation from pathogenesis (i.e., what causes disease) to the origins of health, coping, and resilience [34]. For individuals living with a chronic condition like RA, well-being is often developed through adapting to daily challenges and finding meaning, rather than realizing a complete absence of symptoms. Salutogenesis is particularly highly relevant to this context through its core concept of a Sense of Coherence (SOC), which highlights comprehensibility, manageability, and meaningfulness [34,35]

This study views ceramics not simply as a leisure activity, but as a potential way to help people to enhance their SOC. For people in HK living with the unpredictability of RA, the experience of training in ceramics may provide a unique counter-narrative to their illness. Where RA makes the body difficult to control, the clay provides *manageability (control over their bodies and lives)* through mastery of material; where chronic pain can make life feel disordered, the creative process provides *comprehensibility (physical limitations of their disease)* through structure and focus; and where illness can diminish identity, artistic expression rebuilds *meaningfulness (self-expression)*. Guided by the Salutogenic framework, this study examines how shaping clay helps participants to reshape how they view their chronic conditions, demonstrating that this creative process supports resilience by sustaining the self rather than trying to cure the body. This study explores the lived experience of individuals with RA in HK who participate in ceramics. The primary objective is to investigate how engagement in this creative outlet can enhance their SOC, specifically the ways in which this process fosters resilience and improves overall well-being.

## 2. Materials and Methods

### 2.1. Design

Through an interpretative phenomenological approach, this study investigated how people with RA experience the art of ceramics, highlighting the ways in which this creative practice cultivates resilience and enhances QoL. The study employed semi-structured interviews as a primary method; this approach enabled a rigorous and flexible exploration of the subject matter, situated firmly within the participants’ own environments [36].

### 2.2. Participants

This study employed purposive sampling to recruit 16 adults (aged 18 and over) residing in Hong Kong, who were selected based on specific sampling criteria. Eligible people were adults with a confirmed medical diagnosis of RA who were actively participating in ceramics workshops and had completed at least one ceramics workshop course organized by the Hong Kong Arthritis & Rheumatism Foundation between 2024 and 2025. Participant recruitment was achieved upon reaching data saturation. The sample exclusively comprised ethnic Chinese individuals participating in routine medical follow-up, consistent with characteristics of the general local population. Participants diagnosed with RA were recruited for the study through referrals from specialist nurses and local support organizations, such as the Hong Kong Arthritis & Rheumatism Foundation.

### 2.3. Ethical Consideration

With approval from the local academic Research and Ethics Committee, the study was conducted in accordance with the principles of the Declaration of Helsinki and followed all required guidelines (HRE230236). Before the interviews took place, whether in person or over the phone, participants were given a full explanation of the study’s goals and signed a consent form. We made sure that everyone knew that participation was completely voluntary, and that they could walk away whenever they wished. To protect participant privacy, we anonymized the data by removing all identifying information. Additionally, all participants were comfortable speaking Chinese.

### 2.4. Data Collection

Between March and October 2025, a single RA specialist (Y.A.) conducted all Chinese-language interviews to ensure consistency across the study. Data were collected via in-person (*n* = 3) and telephone (*n* = 13) interviews. A dual-mode approach was employed to accommodate the pain, physical constraints, and mobility limitations frequently experienced by individuals with RA, thereby maximizing participant comfort and accessibility. While telephone interviews present potential challenges regarding environmental control, these were mitigated through a strict protocol. Prior to the interview, the researchers confirmed that participants were in a comfortable, private, and quiet home environment, free from noise and other individuals. Existing studies support the use of telephone interviews in qualitative health research, demonstrating that they produce data of comparable depth and quality to face-to-face interviews, particularly when accommodating vulnerable populations with physical limitations [37,38]. Once written consent was secured, every session was captured on audio and then transcribed verbatim. The interviews typically ran between 35 and 100 min. The research team conducted interviews with each participant using a structured guide (Supplementary Table S1). This set of questions was vetted and approved by experts in both psychology and qualitative research methods. The interview guide is available for review. To deepen the analysis, field notes were taken to record details about the environment. Throughout the process, participant safety was the top priority, with the researchers constantly monitoring for distress and ready to offer immediate assistance. In alignment with the interpretative phenomenological approach, data collection and preliminary analysis were conducted together to continuously assess for data saturation. The research team evaluated the depth and richness of the narratives to determine when the essence of the participants’ lived experiences had been fully captured. During the interviews with the final three participants (the 14th, 15th, and 16th), no new perspectives, meanings, or themes regarding the ceramics workshops appeared. Having realized informational redundancy and thematic saturation, data collection was subsequently finalized with a total of 16 participants.

### 2.5. Data Analysis

The individual interview recordings were transcribed verbatim by three researchers (Y.A., T.Z., and Y.J.) utilizing the NVivo software (NVivo Version 12, QSR International, Denver, CO, USA).

#### Reflexivity and Researcher Characteristics

In accordance with the Consolidated Criteria for Reporting Qualitative Research (COREQ) checklist [39], a reflexivity protocol was established to acknowledge and mitigate the influence of the researchers’ professional backgrounds on the study’s design, data collection, and interpretation. The core research team, including the three independent coders, consists of registered nurses with experience in chronic illness care. The wider multidisciplinary research team includes occupational therapists, a psychiatrist, and medical professionals specializing in RA.

Given the predominantly clinical backgrounds of the research team, we recognized a potential preconception: a tendency to view the ceramics workshop primarily through a medicalized or functional lens. For instance, there was a risk of focusing disproportionately on clinical outcomes, such as joint pain, hand mobility, or symptom management, while potentially overlooking the participants’ holistic, psychosocial, or purely creative experiences. To mitigate this bias, the researchers engaged in continuous bracketing throughout the study. The three nurse coders maintained reflexive journals to explicitly document their clinical assumptions prior to and during data analysis. Furthermore, regular multidisciplinary debriefing sessions were held. During these sessions, the diverse perspectives of occupational therapists and psychiatric professionals helped to challenge and balance the strict medical viewpoints. This collaborative, reflexive approach ensured that the participants’ authentic, lived experiences remained central to the analysis and were not overshadowed by the researchers’ clinical preconceptions.

Colaizzi’s seven-step phenomenological method was employed to analyze the data, which included both interview transcripts and field notes [40]. First, the research team immersed themselves in the data by reading it multiple times. This iterative process allowed key themes to surface naturally. To ensure methodological transparency and practice reflexivity, the researchers actively engaged in bracketing their personal biases and preconceptions regarding RA, Chinese cultural norms, and art therapy. This was achieved by maintaining reflexive journals and openly discussing their assumptions prior to and during the analysis. After bracketing, the researchers analyzed the data and organized it into an initial set of codes. The team then refined the codebook through continuous discussion. Ultimately, the final coding framework incorporated these inductive codes to provide a holistic view of the participants’ experiences, revealing the core themes and sub-themes of the study. To ensure the accuracy of the data, the researchers used member checking, having participants review their transcripts to confirm their statements were captured correctly. Concluding the analysis, the team reviewed the raw data once more to validate the accuracy of the final themes. This comparison was essential for assuring that the final structure was not just one person’s view, but a true representation of the combined coding approaches of the whole team.

### 2.6. Rigor

Guided by Colaizzi’s phenomenological framework, three investigators (Y.A., Y.J., and T.Z.) independently analyzed the transcripts to isolate significant statements and obtain themes (Supplementary Table S2). The quality and transparency of this report were further validated through strict adherence to the COREQ checklist (Supplementary Table S3) [39]. The study’s trustworthiness was established using Lincoln and Guba’s framework. To enhance credibility and confirmability, we employed robust triangulation methods. Data triangulation was achieved by cross-referencing the interview transcripts with the observational field notes taken during the ceramics workshops. Furthermore, investigator triangulation was utilized, as the three researchers independently coded the data and performed member checking before convening to reach a team consensus [41]. Detailed follow-up discussions provided clarity on the combining of sub-themes and the discrepancy in interpreting divergent participant narratives. The ongoing authentic lived experiences of the Chinese participants were analyzed.

## 3. Results

Sixteen Chinese patients with a confirmed diagnosis of RA and a history of regular clinical follow-up were recruited for this study (Table 1). The themes and sub-themes of this study are summarized in Table 2.

### 3.1. Theme 1: Embodied Manageability: The Art of Negotiating Ability Within a Changing Body

Despite the visible physical features of their illness, such as fingers that have drifted and stiffened into new, uncompromising shapes, most participants reported surprising autonomy within the clay. Where participants might expect the deterioration of fine motor skills and the constant whisper of chronic pain to act as overwhelming barriers, the ceramics workshop became a space of alteration rather than limitation. They described a process of intuitive adaptation; if a thumb could not press, a palm could smooth; if a joint could not bend, the weight of the arm could be leveraged. This was not a denial of their disfiguration or pain, but a tactile reclaiming of agency, where the very hands that often felt like betrayers in daily life became capable tools of creation once again, proving to themselves that their ability to shape the world around them had not been lost, only translated into a different language of touch.

*“My fingers, stiff and disfigured by illness, often feel like betrayers in daily life, causing me to avoid even simple tasks. Yet, touching the cool, smooth clay felt surprisingly forgiving. Guided by using tools rather than forcing my unbending joints, I navigated through the chronic pain. Watching a pot take shape, I was struck by a deep sense of disbelief and pride. Home highlights my limits, but here I found I can still create on my terms.”* (P03)

While participants had accepted the decline in their hand and finger function due to RA, they made a concerted effort to participate in the entire ceramics workshop. They tried to maintain their abilities by carefully following the instructor’s guidance throughout the process.

*“I just finished a 4-session clay workshop, and I am so proud of the result. At first, I was nervous about my thumbs in handling the materials, but I found I could manage the general motion and shaping of the clay quite well. I hit a snag with the smaller details; I could not quite press the little pieces of clay with enough precision on my own. Thankfully, my instructor was amazing, she stepped in to assist me with those challenging parts while I followed her lead step-by-step. It’s honestly amazing to look at this finished product and say, ‘I did this!’”* (P13)

Demonstrating remarkable perseverance, several participants managed to push past their physical joint pain and the challenges of commuting to the venue in order to fully engage in the ceramic workshops.

*“I am proud that I managed to travel by myself on the MTR (Mass Transit Railway) to the Nam Shan Estate venue for every single session of this ceramics workshop. Being diagnosed with RA makes travel difficult for me, the walk from the station is quite long, and the inclined roads are real challenge to walk up. It definitely took me some extra time to get there, and I was often in pain, but I did not let that stop me. I stayed involved for all the sessions because I wanted to prove to myself that I could manage it.”* (P14)

*“Even though it took a long time, I managed to walk from the Kai Tak MTR station to the class entirely on my own, leaning on my walking stick the whole way. I feel so helpless when it rains. My hands just won’t cooperate enough to open an umbrella, leaving me completely soaked. Then I’ll get trapped outside because I don’t have the strength to pull open a heavy door. But honestly, even though every step hurts, I was determined not to let that stop me from getting to class.”* (P02)

### 3.2. Theme 2: Clear Comprehension of Body Limits: A Pressure-Free, Supported Creative Experience

For participants, the ceramics workshop became a pressure-free environment. As they molded clay, they gained a clear, experiential understanding of the movement limitations in their hands and fingers. However, seeing their self-made products fostered pride rather than frustration. This was not just art; it was holistic self-management guided by healthcare professionals and instructors. When combined with medication and empowering home clay exercise, this creative engagement allowed participants to safely accept their physical boundaries while discovering new strengths in a supportive environment.

*“The studio felt safe because we were all in the same boat. As I shaped my cup, I noticed my nieghbor had strong fingers, while the person aacross from me had joints that were quite twisted and weak. But that difference in our hands made me feel free. There was no competition here, just a quiet understanding of our bodies’ limits. I did not need to apologize for my slow hands or unbending fingers. I found pure joy in working at my own rhythm without rushing. Withh no burdens to carry, I finally felt free, just me, the clay, and the space to be myself.”* (P15)

*“I joined every ceramics session feeling completely free of pressure, allowed my mind to settle into a truly positive space. There was never any need to compare myself to others, allowing me to simply focus on my own experience without judgment. I found myself pausing in a moment of deep mental stillness, gathering my thoughts before I touched the clay.”* (P06)

While many participants found the tactile nature of clay empowering, divergent cases revealed the stark limitations of the intervention, particularly when confronted with the irreversible physical damage caused by RA. For instance, one participant expressed a sense of resignation rather than relief, stating.

*“I only wanted to finish the whole lesson, it cannot heal the disfigurement of my fingers.”* (P16)

Participants stated that continuous hand movement lessened stiffness and strengthened flexibility during the workshop. Additionally, they utilized occupational therapist-guided home clay exercises as a valuable supplementary strategy for symptom management.

*“The two-hour session offered a unique opportunity for continous hand exercise I do not get in daily life. Despite a little joint pain, I kept going because the non-stop movement actually helped. My morning stiffness, typical of my RA, weakened as my fingers became more flexible through the constant action. Beyond the workshop, therapist provided clay and videos for home practice. These supplementary exercise are absolutely important, helping me actively manage my symptoms and maintain that flexibility.”* (P05)

The workshop instructor, also an RA patient, led participants’ intense focus on the clay. Her step-by-step support removed disturbances, securing the successful completion of the finished product.

*“I found myself getting completely absorbed in the physical act of pushing the clay. It helped that my instructor also RA, she truly understood what I’m going through physically. She walked every classmate through the process step-by-step until the piece was finished. Her approach really helped us tune out distractions little by little, until we’re fully focused on achieving exactly what we wanted for that lesson.”* (P07)

### 3.3. Theme 3: The Meaningfulness of Creating Art: Gaining Confidence Despite Physical Limits

Participants living with RA discovered deep meaningfulness and renewed self-worth through participation in a ceramics workshop. Despite facing significant physical barriers, including joint pain, hand disfiguration, and decreased upper limb strength, these participants successfully adapted their movements to create unique art products. The finished ceramic pieces served as palpable proof of their adaptability, generating a deep sense of satisfaction that contrasted sharply with the limitations they experienced in daily self-care. This creative process facilitated a psychological shift from feelings of deterioration and dependence to a state of joy and restored confidence. The impact of this transformation extended beyond the individual, positively influencing their family dynamics. By redefining themselves as creators rather than only patients, participants demonstrated that despite the physical destruction of RA, they possess the capacity to generate beauty, pride, and personal meaning.

*“Mornings usually start with me unable to button my shirt. My fingers are twisted, making me feel defeated. But the clay was forgiving. When my fingers would not bend, I used the hell of my palm to shape the earth. The pain became part of creating, not just suffering. When I brought my bowl home, my husband did not look at my swollen joints with empathy, he looked at the art with wonder. I realized I’m not just a deteriorating body. I created something beautiful out of struggle. Finally, I felt useful again.”* (P04)

*“The hardest part was not pain, but weakness. My unsteady movement arms just lifting a pot. Working the clay was a battle, but it was a ‘good pain’, pain with a purpose. Since I lacked arm strength, I leaned my whole-body weight into the table, adapting to my limits. Holding my finished vase was holding proof of my power. When I showed my son, he did not rush to help me, he just smiled with pride. For so long, my life was just about managing my condition. Now, I feel like I’m finally leaving my own mark on the world.” (P09)*

The participants expressed a deep desire to continue with future sessions, highlighting how much they enjoyed the freedom and unpredictability of creating. They reported that seeing their finished pieces provided an intense sense of satisfaction and personal achievement.

*“I eagerly anticipate every single class. There’s such freedom in letting the clay guide my hands in the moment, and seeing the final piece fills me with an overwhelming, undeniable sense of achievement. I found a deep, quiet sense of calm settling over me when I’m in that space.”* (P01)

Following occupational therapists’ assessment in the final session, the participants observed a slight improvement in finger strength compared to the start of the program. They expressed that they did not anticipate extreme changes, but found this small noticeable increase to be sufficient, receiving a deep sense of satisfaction from the concrete evidence of their physical progress.

*“I honestly did not think it was possible with my hands like this, but I proved myself wrong. I actually can do it. I’m stretching myself further than I ever thought possible. To me, every artwork I finish was invaluable.”* (P02)

### 3.4. Theme 4: Supporting Resilience: Peer Exchange and Holistic Self-Management Strategies

Participants in the ceramic workshops described a heartfelt sense of resilience that extended far beyond the physical act of molding clay. While recognizing that art is not a cure for RA, participants valued the studio as a key space for nurturing their well-being and practicing self-management. The studio naturally expanded to a supportive community where participants shared both creative techniques and medical advice. Conversations seamlessly transitioned from art to health management, covering topics like biologic treatments and Traditional Chinese Medicine (TCM) practices, including acupuncture, massage, and Tai Chi. Influenced by this supportive environment, many participants adopted extra physical routines to prevent mobility loss. They began integrating gym practices and home exercises alongside their ceramics work to specifically strengthen their limbs. Finally, participants noted a marked improvement in their condition. They believed in their renewed sense of agency through the powerful combination of creating art, learning from one another, and taking proactive steps to maintain their physical strength.

*“Honestly, I did not walk in here under the illusion that throwing a pot would be some magic cure for my joints. I’m realistic about the damage RA does. But this workshop has become the anchor for how I manage everything else. It’s the conversations in between the glazing that really matter. Just last lesson, while we were squeezing clay, the woman next to me explained how she managed the side effects of her biological injects, and it was advice I could not get from my doctor (rheumatologist). It pushed me to stop being passive. Now, I do not just sit at home. I’ve started hitting the gym on the days I’m not here to keep my grip strength up. It’s about maintenance. I treat this class, the gym, and my meds as a single package. My hands might still hurt, but I feel stronger and more in control than I have in years.”* (P10)

Participants’ gained support in community sharing; they integrated clay work with holistic practices like Tai Chi. This gentle, combined approach restored their spirit and agency.

*“There is a quiet healing here that goes deeper than just the physical body. When I’m at the wheel, the pain declined into the background, but the real comfort came from the circle of friends I’ve found. We share our burdens so openly. I remembered feeling so hopeless about my stiffness until a classmate shared her journey with Tai Chi and acupuncture alongside her regular treatment. It opened a door for me. I realized I could be gentle with myself while still fighting back. Now, I blend my home exercises with clay work, treating my movement like a form of meditation. I felt a shift inside me, a lightness. It’s not that the disease was gone, but my spirit was not crushed by it anymore, I’m molding my life just like I mold the earth.”* (P09)

Treating clay as a living entity, participants assumed a slower, deliberate pace. The creative process effectively transformed impatience into a steady, mindful practice.

*I’ve discovered that clay sets its own pace. It pushes past my impatience, requiring slow, unhurried pressure to shape anything successfully. Finally, it’s a beautiful lesson in patience, making me take everything one careful moment at a time.”* (P11)

A major takeaway for the participants was how deeply they evaluated the sense of community and peer encouragement that grew out of the classes. They expressed that these ongoing connections provided necessary encouragement and a sense of consensus in facing their condition.

*“This workshop was a limited gift because it connected me with people who are fighting the same illness. We’ve stayed close, meeting up to eat and trade information about treatments and our experiences. That shared empathy is an enormous source of strength, making the isolating journey of rheumatism feel a lot less lonely.”* (P08)

## 4. Discussion

This study examined how Chinese RA patients experienced ceramics workshops, aiming to understand their unique daily challenges and explore how artistic practice promotes resilience and overall well-being. Drawing on Antonovsky’s Salutogenic theory and the framework of SOC, this study explores how people navigate the difficult reality of joint pain and declining mobility [34,35]. To demonstrate rigorous theoretical mastery and move beyond anecdotal understanding, our identified themes can be explicitly mapped onto Antonovsky’s three pillars of health: comprehensibility, manageability, and meaningfulness [42].

First, comprehensibility appeared through understanding *the body’s limits when shaping*. Through their interaction with the clay, participants developed a clearer cognitive awareness of their physical boundaries, making the unpredictable nature of RA more predictable and understandable in a spatial and physical context. Second, manageability was realized through the “*embodied manageability*” *of clay*. Participants illustrated a high level of agency, utilizing the tactile, forgiving nature of the medium to regain a sense of physical control [34,35]. They actively merged their chronic condition and declining mobility into their routines, demonstrating that the physical demands of clay work could be adapted and managed [43,44,45]. Finally, meaningfulness was profoundly evident in *the significance of creating art*. Despite their physical limitations, patients sustained their well-being by finding deep emotional, cultural, and personal value in the act of artistic creation, which motivated them to overcome the hurdles of their illness.

By explicitly connecting our findings to these three pillars, the study demonstrates that participants can sustain their well-being by viewing their circumstances as understandable, manageable, and meaningful, three crucial factors in building resilience [42]. Therefore, this study was conducted from an interpretative perspective. If we fail to consider these specific cultural characteristics and theoretical mechanisms, we risk creating major barriers for this community down the line [34,35]. To deliver truly effective care, healthcare providers must grasp the underlying Salutogenic processes that help patients to move from a state of vulnerability to one of resilience; otherwise, their support may fall short [43,45]. To ensure that community-based support initiatives receive the funding they desperately need, it is required that we first bridge this gap in our understanding. In this way, we can establish reliable, accessible, and high-demand community activities, such as ceramic workshops, that truly meet the needs of RA patients [46,47,48].

Theme 2 delineates that the workshops provided a shelter from pressure, significantly enhancing comprehensibility regarding the participants’ condition. By facing their pain, physical limitations, and daily symptom shifts within a supportive environment, participants were empowered to transform their experience from a huge burden into one of conscious adaptation and self-awareness [43,46,49,50]. The sessions facilitated discussions on emotional well-being and fostered a sense of satisfaction through ceramic creation, specifically the freedom to handle clay without demand. These findings reflect the complex reality of managing RA daily and underline how the structured nature of the workshop helped these vulnerable patients to clearly comprehend and respect their bodily limits [46,51]. The intervention uniquely integrated creative expression with clinical care by incorporating regular check-ins into the workshop, allowing occupational therapists to systematically track hand and finger strength in a non-clinical setting. To ensure that progress continued outside the studio, therapists equipped participants with exercise videos and a home practice plan. Participants acknowledged this professional support, but emphasized that their motivation was built on a comprehensive circle of trust, including peers and family. Furthermore, the emotional support provided by family members at home likely strengthened participants’ motivation, enabling them to sustain their progress and apply it to their daily lives [52,53]. This support system turned home clay practice into a successful new habit, helping patients to bridge the gap between good intentions and actual physical conditioning.

Theme 3 highlights that the workshops provided deep personal meaning, going beyond practical self-management. This finding is especially significant for patients of Chinese families, as their cultural norms often highlight internalizing emotions over verbal expression, making alternative directions for emotional processing particularly valuable [54]. This reluctance to vocalize personal struggles frequently prevents these individuals from engaging with standard, talk-centric support groups, emphasizing a critical gap in conventional care models. Therefore, tailored activities like ceramics are recommended over standard patient education. The study suggests that interventions concentrating on intrinsic processes are most successful; the deep concentration required to craft artistic works allows patients to develop an internal locus of control [43,55]. The visible satisfaction of completing functional items, such as cups, plates, bowls, and pots, reinforces this positive behavior and fosters a sense of achievement.

In terms of supporting resilience, for the participants success was not defined by an immediate physical cure, but by the sustained effort to manage their well-being and slow the deterioration of their movement [42,43]. The support group also acted as a critical platform for knowledge exchange, allowing patients to share various treatment updates that encompassed both modern pharmacological advancements, such as biologics, and traditional approaches like TCM. Participants also shared lifestyle changes, such as joining gyms, practicing Tai Chi, or integrating specific training exercises. The patients’ reliance on an integrative approach that blends Western and TCM with proactive lifestyle modifications emphasizes a strong demand for community-based workshops designed to support holistic self-management and prevent physical degeneration [56,57]. By employing inductive coding for data that did not fit pre-existing categories, the research team identified the critical importance of resilience and self-management in patients with RA.

RA generally affects women, which partly explains the gender skew in our participant sample; however, the specific intersection of age, gender, and Chinese cultural background significantly shaped the participants’ experiences. For example, older Chinese women may hold specific cultural views regarding chronic illness, acceptance, and traditional gender roles, which could influence how they are involved with and gain meaning from a ceramics workshop [28]. The therapeutic benefits observed, such as stress reduction, identity reconstruction, or social connection, might be experienced or expressed alternatively by younger patients, male patients, or individuals from different cultural backgrounds. Accordingly, while this study offers deep insights into this specific demographic experience, the findings cannot be broadly generalized to the wider RA population. Furthermore, individual interviews allowed participants to detail their personal experiences, which facilitated a comprehensive and holistic understanding of their specific care needs. Future quantitative research approach should aim to include more heterogeneous cohorts; clinical measurements of joint deformity for formal comparison; and populations containing younger patients, males, and individuals from diverse ethnic and cultural backgrounds, in order to explore how different demographic factors affect the therapeutic efficacy of art-based interventions like ceramics.

### Limitations

While this study anticipates valuable insights into the benefits of integrative peer support, the findings should be interpreted considering several methodological constraints. A primary limitation of this study is the small and highly homogeneous sample, which consisted mainly of Chinese females aged 60 and above. While small sizes are standard and appropriate in qualitative phenomenological research, where the goal is to obtain a deep, rich understanding of lived experiences rather than statistical representation, the demographic regularity limits the generalizability (or transferability) of the findings. Furthermore, the recruitment strategy presented a notable selection bias. Because participants were recruited from ceramics workshops, it is highly likely that they already had a pre-existing interest in art or a positive susceptibility toward creative activities. This self-selection may have led to overly positive findings regarding the intervention’s therapeutic benefits. Patients who are uninterested in ceramics, or those experiencing severe RA symptoms, such as intense fatigue or severe joint pain in the hands that prohibit them from participating, are not represented in this study.

First, the study was conducted with a relatively small cohort of 16 participants. Because the sample was exclusively recruited from ceramics workshops in HK, the findings may be closely tied to this specific cultural and environmental context, limiting their replicability to larger populations or different therapeutic settings. As a result, these findings may lack generalizability to the wider Chinese population or to male patients with RA. Because our cohort was entirely female and predominantly older, it does not fully capture the demographic diversity of those living with the disease. The demographic consistency presents an inherent selection bias. Consequently, the unique challenges and perspectives of younger patients, as well as those from diverse socioeconomic backgrounds, may be underestimated in our findings. Furthermore, cultural nuances shaped the data collection process. The Chinese cultural stress on politeness and social harmony, often characterized by a strict separation between internal emotions and outward confidence, may have inhibited participants from openly revealing their true frustrations or negative experiences. Participants might have felt social pressure to appear appreciative or impassive, leading them to withhold their true needs regarding advanced medical interventions, such as biological agents or newer treatment modalities, in favor of discussing the psychosocial benefits of the workshop.

## 5. Conclusions

This qualitative study demonstrates that, while individuals with RA exhibit remarkable personal agency and resilience, targeted external support remains essential in empowering them to navigate and transcend their physical limitations. Although ceramics workshops served as a highly effective medium for fostering this empowerment, our findings highlight a broader need for formalized peer support programs within chronic care settings. The participants’ lived experiences reveal a strong demand for comprehensive support networks that extend beyond standard social interaction. Such programs should provide a dual platform that facilitates medical information exchange while offering engaging, meaningful activities designed to elevate QoL. Therefore, further healthcare interventions must prioritize the cultivation of these peer networks. By generating structured opportunities for patients to share practical self-management strategies, healthcare providers can better equip them to handle the complex emotional, medical, and social realities of living with RA on a daily basis.

## Figures and Tables

**Table 1:** Characteristics of participants.

	Mean (Min–Max)	n	(%)
Age	61.6 (from 40 to 73)		
Marital status			
Single		6	(38)
Married		5	(31)
Divorced		4	(25)
Widow		1	(6)
Education level			
Primary		0	(0)
Secondary		10	(62)
High diploma		3	(19)
Bachelor’s degree or above		3	(19)
Employment			
Full-time		2	(13)
Part-time		5	(31)
Retired/unemployed		9	(56)
Years since rheumatic arthritis diagnosis			
3–9		6	(38)
10–16		5	(31)
17–23		3	(19)
24–30		1	(6)
>31		1	(6)
Treatments			
Medication		7	(44)
Medication + Biological Agent		1	(6)
Medication + TCM *		4	(25)
Biological Agent + TCM *		1	(6)
Medication + Biological Agent + TCM *		3	(19)
Participated in various arts and crafts activities prior to attending ceramic workshops			
Yes		4	(25)
No		12	(75)
Religion			
Buddhist		4	(25)
Christian		5	(31)
Catholic		1	(6)
Nil		6	(38)

* TCM: Traditional Chinese Medicine consists of acupuncture, Chinese therapeutic massage, and Tui Na.

**Table 2:** Themes and sub-themes of this study.

	Themes	Sub-Themes	Number of Participants’ Quotes
1	Embodied manageability: The art of negotiating ability within a changing body	- Adapting physical techniques to joint stiffness and pain - Accepting bodily unpredictability to reclaim a sense of control	16
2	Clear comprehension of body limits: A pressure-free, supported creative experience	- Recognizing physical boundaries and learning to pace - Benefiting from a non-judgmental and supportive environment	14
3	The meaningfulness of creating art: Gaining confidence despite physical limits	- Transforming the experience of pain into tangible creation - Rediscovering self-worth and shifting identity through artistic completion	12
4	Supporting resilience: Peer exchange and holistic self-management strategies	- Finding solidarity and exchanging practical coping mechanisms with peers - Integrating creative experience into holistic daily self-care	13

## Data Availability

The data that support the findings of this study are available from the corresponding author upon reasonable request.

## Supplementary Materials

The following supporting information can be downloaded at: https://www.mdpi.com/article/10.3390/healthcare14081069/s1, Table S1. Interview Discussion Guide. Table S2. Strategies for Establishing Trustworthiness. Table S3. COREQ Checklist.

## Abbreviations

The following abbreviations are used in this manuscript:

COREQ	Consolidated Criteria for Reporting Qualitative Research
HK	Hong Kong
SOC	Sense of Coherence
QoL	Quality of Life
TCM	Traditional Chinese Medicine
