# Supplementary material for: Molding the Pain into Porcelain: The Silent Resilience of Arthritic Hands in Hong Kong’s Ceramic Studios
Source: Healthcare (Basel). 2026 Apr 17;14(8):1069. doi: 10.3390/healthcare14081069 (PMC13115973; doi:10.3390/healthcare14081069)
Supplement: Supplementary file 1 [file healthcare-14-01069-s001.zip › healthcare-4217942-supplementary.pdf]

Supplementary Table S1. Interview Discussion Guide.

1. Why did you decide to start going to ceramics classes in the first place?
2. Take me through a normal day in the studio. What do you usually do from start to finish?
3. How do these workshops affect you on a personal level? For example, have you noticed any changes in your mood, your mindset, or how you connect with others, either while you're in the studio or after you go home?
4. Have you encountered any challenges or difficulties related to your rheumatoid arthritis when engaging in ceramics workshop? If so, how did you address these?
5. How do you feel doing ceramics has influenced your quality of life or your day-to-day experience with rheumatoid arthritis?

Supplementary Table S2. Strategies for Establishing Trustworthiness.

Indicators of Rigor	Strategies	Ways to strengthen the study's results	Practical implications
Credibility	To enhance the credibility of the analysis	To conduct an in-depth, longitudinal exploration of participants' experiences through multiple analytic lenses	- To establish the credibility of our findings, we utilized the strategy of prolonged engagement. Conducting multiple sessions with participants fostered the trust necessary to mitigate social desirability bias; a prevalent challenge withing Hong Kong's health-conscious culture. Consequently, we captured an authentic representation of their daily habits and lifestyles, resulting in rich, authentic data<br>- To enhance the credibility of our findings, we employed data triangulation by integrating interviews, personal photo-diaries, and ceramic products. This multifaceted approach allowed us to uncover discrepancies between participants stated intentions and their actual behaviours. For example, while many participants reported strict adherence to Western medicine, their diaries revealed frequent engagement with gym exercises and Traditional Chinese Medicine (TCM) as complementary treatments. Revealing these underlying tensions ensures that our findings authentically reflect the complex, pluralistic reality of healthcare practices in Hong Kong
		To engage participants in rich, exploratory dialogues	- Research team prioritized a systematic refinement of the interview guide following ethical approval to guarantee data quality and validity. During two dedicated team meetings, the questions were subjected to optimize clarity and verify their alignment with our overarching research goals<br>- Following the peer review, three pilot interviews were administered to assess the interviews flow and question comprehensibility. As these sessions demonstrated the efficacy of the interview guide without necessitating any revision, the pilot data were retained for the main analysis
		To ensure credibility by grounding the findings firmly in the participants' perspectives	- Our research team included members with both formal academic backgrounds and practical, hands-on experience in qualitative research<br>- To ensure consistent, high-quality data collection, the team was mentored and supervised by a senior researcher with over seven years of experience
		To safeguard the study's trustworthiness by retaining all primary data for potential external audits	- Field notes capturing non-verbal cues and environmental contexts were recorded during interviews and analysed concurrently with the transcript. This dual approach deepened our contextual understanding of the narratives and enhanced the study's dependability

		To establish the credibility of the analysis through ongoing peer scrutiny, ensuring that emerging themes are rigorously challenged and refined	- To maintain analytical rigor, the research team conducted bi-weekly peer debriefing sessions with expert Fellows from the Hong Kong Academy of Nursing and Midwifery. These consultations provided an essential external perspective, enabling us to critically evaluate our emerging interpretations and identify any overlooked nuances in the data
Dependability	To confirm the consistency of the data interpretation	To delineate the methodological approach with comprehensive detail and contextual clarity	- By detailing their methodological procedures comprehensively, the selected articles enabled the tracing of an audit trail, which bolstered the dependability of their conclusions<br>- By keeping a thorough record of all our data collection steps, the whole team helped guarantee the dependability of the study
		To ensure the study's dependability by keeping a rigorous log of the research trajectory	- The research team established credibility via member checking, inviting participants to review interview syntheses and authenticate that our emerging interpretations captured the reality of their lived experiences
		To support the study's dependability through the meticulous maintenance of an audit trail detailing all methodological decisions	- To establish methodological rigor and dependability, we utilized a consensus coding strategy. This ensured that all team members applied the codebook uniformly, safeguarding the analysis against individual biases
Transferability	To facilitate the transferability of the research to similar groups or environments	To collect data until data saturation is achieved and informational redundancy is reached	- - The research team concluded data collection upon reaching data saturation, defined as the point of informational redundancy where additional interviews no longer produced new codes or elaborated on established themes<br>- Th depth of the participants' accounts facilitated a thick description, supporting a highly contextualized interpretation of their lived experiences with rheumatoid arthritis
Confirmability	To maintain objectivity and establish an audit trail, ensuring the findings are easily confirmable	To apply in critical reflexivity regarding the research process	- The research team fortified the study's methodological rigor through a combination of personal reflexive journals and bi-weekly peer debriefing, ensuring a consistent and critically reflexive analytical process

Supplementary Table S3. COREQ Checklist.

COREQ (COnsolidated criteria for REporting Qualitative research) Checklist

A checklist of items that should be included in reports of qualitative research. You must report the page number in your manuscript where you consider each of the items listed in this checklist. If you have not included this information, either revise your manuscript accordingly before submitting or note N/A.

Topic	Item No.	Guide Questions/Description	Reported on Page No.
**Domain 1: Research team and reflexivity**			
Personal characteristics			
Interviewer/facilitator	1	Which author/s conducted the interview or focus group?	Title page
Credentials	2	What were the researcher's credentials? E.g. PhD, MD	Title page
Occupation	3	What was their occupation at the time of the study?	Title page
Gender	4	Was the researcher male or female?	NA
Experience and training	5	What experience or training did the researcher have?	title page, 3-5
Relationship with participants			
Relationship established	6	Was a relationship established prior to study commencement?	3-5
Participant knowledge of the interviewer	7	What did the participants know about the researcher? e.g. personal goals, reasons for doing the research	3-5
Interviewer characteristics	8	What characteristics were reported about the inter viewer/facilitator? e.g. Bias, assumptions, reasons and interests in the research topic	3-5
**Domain 2: Study design**			
Theoretical framework			
Methodological orientation and Theory	9	What methodological orientation was stated to underpin the study? e.g. grounded theory, discourse analysis, ethnography, phenomenology, content analysis	3-5
Participant selection			
Sampling	10	How were participants selected? e.g. purposive, convenience, consecutive, snowball	3-5
Method of approach	11	How were participants approached? e.g. face-to-face, telephone, mail, email	3-5
Sample size	12	How many participants were in the study?	3-5
Non-participation	13	How many people refused to participate or dropped out? Reasons?	NA
Setting			
Setting of data collection	14	Where was the data collected? e.g. home, clinic, workplace	3-5
Presence of non-participants	15	Was anyone else present besides the participants and researchers?	NA
Description of sample	16	What are the important characteristics of the sample? e.g. demographic data, date	5-6
Data collection			
Interview guide	17	Were questions, prompts, guides provided by the authors? Was it pilot tested?	Title page, 3-5
Repeat interviews	18	Were repeat inter views carried out? If yes, how many?	NA
Audio/visual recording	19	Did the research use audio or visual recording to collect the data?	3-5
Field notes	20	Were field notes made during and/or after the inter view or focus group?	3-5, suppleme
Duration	21	What was the duration of the inter views or focus group?	3-5
Data saturation	22	Was data saturation discussed?	3-5, supplemer
Transcripts returned	23	Were transcripts returned to participants for comment and/or correction?	3-5, Supplemer
**Domain 3: analysis and findings**			
Data analysis			
Number of data coders	24	How many data coders coded the data?	5-9
Description of the coding tree	25	Did authors provide a description of the coding tree?	NA
Derivation of themes	26	Were themes identified in advance or derived from the data?	5-9
Software	27	What software, if applicable, was used to manage the data?	4
Participant checking	28	Did participants provide feedback on the findings?	3-5
Reporting			
Quotations presented	29	Were participant quotations presented to illustrate the themes/findings? Was each quotation identified? e.g. participant number	5-9
Data and findings consistent	30	Was there consistency between the data presented and the findings?	5-9
Clarity of major themes	31	Were major themes clearly presented in the findings?	5-9
Clarity of minor themes	32	Is there a description of diverse cases or discussion of minor themes?	5-9

Developed from: Tong A, Sainsbury P, Craig J. Consolidated criteria for reporting qualitative research (COREQ): a 32-item checklist for interviews and focus groups. *International Journal for Quality in Health Care*. 2007. Volume 19, Number 6: pp. 349 – 357

Once you have completed this checklist, please save a copy and upload it as part of your submission. DO NOT include this checklist as part of the main manuscript document. It must be uploaded as a separate file.
